# Analysis on Population Level Reveals Trappability of Wild Rodents Is Determined by Previous Trap Occupant

**DOI:** 10.1371/journal.pone.0145006

**Published:** 2015-12-21

**Authors:** Marc J. Brouard, Tim Coulson, Chris Newman, David W. Macdonald, Christina D. Buesching

**Affiliations:** 1 E2D Ecological and Evolutionary Dynamics, Department of Zoology, University of Oxford, Oxford, Oxfordshire, United Kingdom; 2 Wildlife Conservation Research Unit, Department of Zoology, University of Oxford, Tubney, Oxfordshire, United Kingdom; University of Missouri Kansas City, UNITED STATES

## Abstract

Live trapping is central to the study of small mammals. Thus, any bias needs to be understood and accounted for in subsequent analyses to ensure accurate population estimates. One rarely considered bias is the behavioural response of individuals to the trap, in particular the olfactory cues left behind by previous occupants (PO). We used a data set of 8,115 trap nights spanning 17 separate trapping sessions between August 2002 and November 2013 in Wytham Woods, Oxfordshire, UK to examine if the decision to enter a trap was affected by the PO, if this was detectable in traditional Capture-Mark-Recapture trapping data (i.e., individuals not uniquely marked), and if it was possible for this effect to bias the population estimates obtained. Data were collected on *Apodemus sylvaticus*, *Myodes glareolus*, and *Microtus agrestis*. Three Generalised Linear Models revealed a significant tendency for the three species to enter traps with same-species PO. With, for example, *A. sylvaticus* 9.1 times more likely to enter a same species PO trap compared to one that contained a *M. agrestis* in the grassland during the nocturnal period. Simulation highlighted that, when all other factors are equal, the species with the highest PO effect will have the highest capture rate and therefore return more accurate population estimates. Despite the large dataset, certain species-, sex-, and/ or age-combinations were under-represented, and thus no effects of any additional individual-specific characteristics could be evaluated. Uniquely marking individuals would allow for the PO effect to be disentangled from other biases such as trap-shyness and spatial heterogeneity, but may not be possible in all cases and will depend on the aims of the study and the resources available.

## Introduction

Small mammal community dynamics are typically studied using single capture live traps [1]. Many small mammals live in the leaf-litter or below ground and are nocturnal. Consequently live-trapping is the most effective way to generate population estimates. Live trapping is, however, not a random sampling technique [2]. Yet many traditional methods of estimating population densities (Minimum Number Alive [3], Lincoln-Petersen Index [4, 5]) rely on an equal chance of capturing each species (or sex) present inside the trapping grid [4, 6–8]. Methods have been developed to account for some of these issues [9–11], but they require for each animal to be marked uniquely. Nevertheless as marking each animal uniquely can be logistically difficult and costly in terms of resources, it is not always implemented; instead animals are marked to identify recapture only. One potential bias that is rarely considered when looking at rodent communities, is the behavioural responses to the trap, and in particular how the previous trap history affects trappability of particular species or sub-groups (e.g. different sex/age classes of individuals)[12, 13]. Can this process generate sufficient bias to invalidate assumptions about small mammal community dynamics based on standard trapping protocols when individuals are not uniquely marked?

Traps are usually set for a number of consecutive nights [14], and checked once or multiple times each day depending on the trap type, grid configuration, habitat, environmental conditions and species [15]. The chance of capturing a particular individual or species, during any given trapping period, is comprised of three components [16] (i) the mechanical effectiveness of the trap being used, (ii) the chance that the trap will be encountered, and (iii) the behavioural response to the trap (trap-shyness/boldness of the individual or species). All three components can generate biases that influence the results obtained from small mammal trapping. The mechanical effectiveness, varying with trap type, can result in variation in trap rates of different species/sub-groups (for review see [17]). The encounter chance can change due to the number of traps placed during the trapping and their spacial positioning in the macro- as well as micro-habitat [18]; as the number of traps encountered by all individuals will not be the same [19, 20]. The heterogeneity of individual-, sex- and species-specific behavioural responses to a trap is governed by a number of factors; some species are neophobic and thus exhibit an aversion to new objects within their range, the temporal length of which can vary among species. To counteract this effect, trap prebaiting, where the trap is locked open for a period of time before trapping, with bait available inside the trap, is often used [21] to allow all individuals in the population to become familiar with the trap [1]. Mounting evidence, however, suggests that familiarity with the trap is not the most important factor determining whether an individual will enter. The olfactory cues in and around a trap can also greatly influence trap entry [22].

It is common for the traps to not be cleaned at each trap check and bedding to be re-used, potentially leaving olfactory cues (faeces, urine-soiled bedding etc) in the traps. Small mammals rely heavily on olfactory cues when foraging, to gain information about their environment [23], conspecifics (including sex) and other species. The utilisation of areas that smell of predators and other competitive species is often reduced [24]. This scent-contamination results in the possibility that recent trapping history can alter the probability of future captures, such as capturing rodents of the same/different sex or species to the previous trap occupant on any given night [13, 16, 25–29]. Olfactory cues provide mammals with numerous details about the scent-donor (i.e. the individual from which the odour originated). The response to olfactory cues left in traps can vary depending on the species [29], sex [30–32], breeding condition [33], age [22], social ranking [12, 16, 34], and deme membership [25] of the previous as well as future occupant(s) of the trap. Conspecific odours can have an effect on the response of which animals enter traps [12, 22, 28, 33–37]. Most studies indicate that conspecific odours are attractive, but it is also possible for conspecific odours to be repellent, where the repellent affect results from the relative social standing of the respective individuals (i.e, subordinate individuals avoid traps scented by dominant animals: [12, 28, 34]). Conversely, the response to inter-species heterospecific odours, varies not only with the species involved [29] but interestingly also with season [32, 38]. Among rodents, the detection of heterospecific odours is involved in socio-spatial geometrics, and can aid in avoiding aggressive encounters between species that compete for habitat resources [32, 39].

Although the effects of odour and their potential impacts on capture success have been studied extensively [40], few studies have investigated large-scale trapping data to uncover the implications of these effect on species community composition and relative abundance. Here we use long-term, low-intensity trapping data collected over 10 years to determine the effects of the species (and sex) of the previous animal occupying a trap on subsequent occupancy, and thus on the derived population estimates, while taking into account other factors (such as habitat, trap time and season) that can impact trappability. We then apply the effects of these biases to mathematical simulations to model how significant/extensive the implications of trap occupancy could be on population estimates.

## Methods

### Ethical Statement

All small mammal trapping and handling undertaken in this study was approved by institutional ethical review (Animal Welfare and Ethical Review Board U1; Department of Zoology; University of Oxford). The Longworth traps used included shrew escape holes, although a Shrew Trapping Licence (under the UK Wildlife & Countryside Act, 1981) was in place for incidental captures. Traps were provisioned with bait, including a source of moisture, placed in shaded areas and checked early morning and evening. Fur clips were used as a sufficient, non-invasive identification method, and thus this work fell below the threshold of the UK Animals (Scientific Procedures) Act, 1986. Work was conducted on an Estate (Wytham) owned by the University of Oxford, and all permissions were in place via the Wytham Research Committee.

### Data collection

Trapping data were collected in Wytham Woods, Oxfordshire, UK (SP462080) during 17 trapping sessions between August 2002 and November 2013, comprising 2 different habitat types (mixed woodland and calcareous grassland), for a total trapping effort of 8,115 trap nights (for details see S3 Table). For a detailed description of the site see [41].

Following the methodology described in [42], trapping was conducted with Longworth traps, equipped with shrew escape holes. Traps were provisioned with hay for bedding and baited with a mixture of grain and hamster food, with carrot or apple as a source of moisture, and traps placed in the open were shaded with vegetation across the nest box. The traps were initially set in the evening and then checked the following morning. They were left set for the period of trapping (either 3 or 4 nights) and checked twice daily, morning and evening (intervals not exceeding 14 hours), giving data for both nocturnal and diurnal activity. Bedding was replaced only if it was damp or in the rare event that the trap had caught a weasel, in which case both bedding and trap were replaced (only 7 weasels were caught over the study period). Individuals were marked with fur clips [1] as a means of identifying either individuals or batches of captures, depending on specific study foci. The same traps were used for all the trapping sessions, traps were only replaced when damaged or worn (<50 over the years). The traps were not left out between trapping sessions; they were cleaned from all bedding, food remains, faeces and urine, and stored in metal boxes with lids but not washed between trapping sessions. The traps were only ever used in the Wytham Woods study area.

Comparisons between species capture probabilities were made using Welch’s unequal variances t-test, with the reported degrees of freedom approximated using the Welch-Satterthwaite equation for the variance estimate.

### Species Models

To understand how the previous occupant (PO) affected subsequent occupants (SO), Generalised Linear Models (GLMs) were fitted to the data; one model for each of the three main species caught, wood mice (*Apodemus sylvaticus*), field voles (*Microtus agrestis*) and bank voles (*Myodes glareolus*). The response variable recorded whether an individual of the focal species had been caught (1) or not (0) in each trap on each trap night. As these data were distributed binomially, a logit link function was employed. Although there is the possibility of three species of PO, field voles were almost exclusively found in the grassland and bank voles in the woodland, thus PO was included in the models as a two level explanatory factor, with the values mouse or vole. The models only included traps that had previously caught a rodent during the current trapping session, and it was assumed that the last occupant in the trap was the one that had the greatest impact on subsequent captures. Additionally the data were subsetted to remove the very rare cases were a bank vole was caught in the grassland or a field vole in the woodland. Month was included in the models to account for any seasonal factors affecting intra-annual variation in trappability such as breeding condition, weather etc. Cumulative number of trap checks was included to account for temporal variation in capture probability (e.g. due to decrease in human scent, habitation / possible reduction neophobic response to traps). To determine if there was any deterioration of the PO effect during the trap session, the number of trap checks since the last capture was also included. Habitat was included as a two level factor for wood mice. All statistical analyses were conducted with R version 3.1.0 [43]. The models with all the explanatory variables and their two-level interactions were fitted to the data and then simplified, by model reduction, until the minimal model was determined [44]. The R predict function was used to derive probabilities for the previous occupant effect, from the three models for both the diurnal and nocturnal periods.

### Sex Models

To assess the impact of PO sex on subsequent trap success, a proportion test (prop.test [43]) was run for the three species. For this analysis, a subset of the data was used to include only trappings where PO and SO involved the same species. It was not possible to include sex in the Species Models because, despite of the large dataset, not enough individuals of each species/sex combination were caught causing certain classes of event to be very rare (for further description of problem see [30]).

### Simulation

To understand under what circumstances the PO effect can bias population estimates, a simulation was coded in Java (version 1.8.0). The parameters for the simulation are listed in S1 Table. Each simulation was run for the woodland habitat and the grassland habitat separately. As above, the simulation only considered wood mice and the one vole species in each habitat type. The parameter values for the simulation were calculated from the data (see Simulation section in Results) and the species models (see Species models section in Results).

The simulation determined the chance of capturing any uncaught individuals in a given trap based on the parameter values. Therefore capture chance for an uncaught individual was based on its species (*M*_*c*_ or *V*_*c*_), and if there was a PO in the trap. In this case a multiplier was applied to the value depending on if the PO was of the same or different species (*M*_*s*_, *M*_*d*_, *V*_*s*_ or *V*_*d*_). Finally this value was divided by the total number of individuals in the population of the same species as the uncaught individual (*N*(*mice*) or *N*(*voles*)). This then gave the final capture chance for the individual. To determine which, if any, individual was caught in a trap, Java’s TreeMap class was used [45]. The process can be visualised as a bar that runs from 0 to 100, where the available individuals are placed along the bar, with the amount of bar they occupy determined by their capture chance. The system then randomly chooses a location along the bar, if an individual occupies that location, then it is caught, otherwise no capture is made. For a formalisation of the simulation, see S1 Text.

To understand how the PO effect influences capture rate, with differing numbers of each species in the population, the simulation runs were repeated twice. First with an equal number of each species (20 mice and 20 voles), and second with unequal numbers, 20 mice to 5 voles for the woodland habitat and 20 mice to 80 voles in the grassland habitat. In both cases the number of traps was set to 50. As population estimates derived from our data set could be biased by the PO effect, the species population numbers used above were estimated from the literature [46], with 50 traps representing a trapping area of approximately half a hectare. The capture proportions were then compared, with capture proportion being defined as the number of individuals of a particular species caught divided by the total number of individuals of both species caught during the trapping session. The expected capture proportion for voles $E_{v}=\frac{N(voles)}{N(voles)+N(mice)}$ was compared to the vole capture proportion from the simulation $S_{v}=\frac{n(volescaught)}{n(volescaught)+n(micecaught)}$ using a one-sample t-test. The simulated capture proportion for voles *S*_*v*_ was also compared against the expected capture proportion based on the capture chance without any previous occupant effect $C_{v}=\frac{V_{c}}{V_{c}+M_{c}}$ using a one-sample t-test.

Finally, to determine how differing levels of the PO effect can bias the estimates from small mammal studies, the simulation was run multiple times with both species being set to the same constant parameters values except for the mouse same species multiplier which was varied from 0.1 to 4 by increments of 0.01. The capture chance for both species was set to 20% and the other PO multipliers set to 1, with 50 traps over 5 trap checks and 50 individuals of both species.

## Results

**Fig 1:**
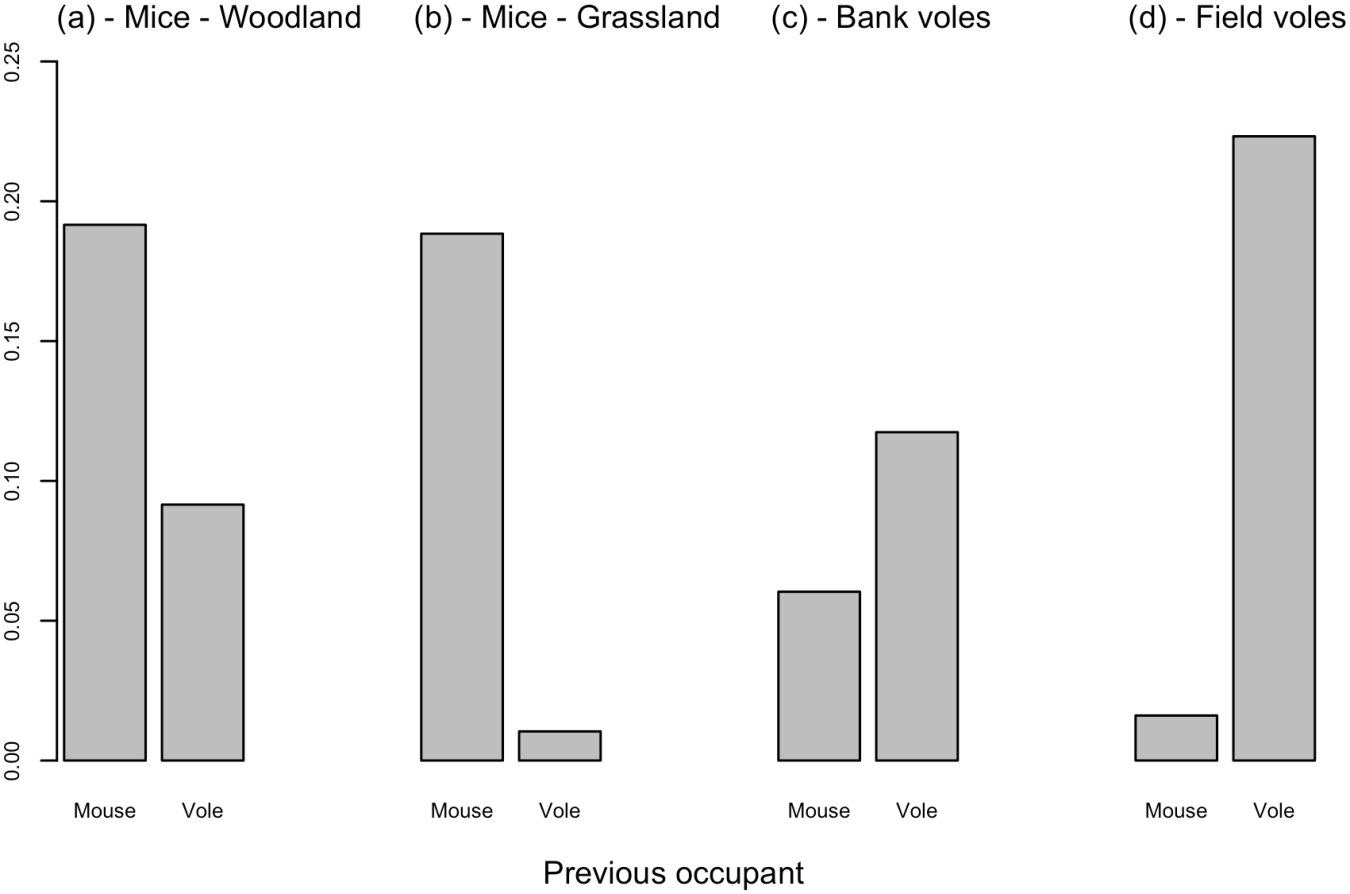
The proportion of each species caught. Depending on the previous occupant in the trap. The values were calculated from the raw data.

During the study period, a total of 1,343 traps were set, comprising 8,115 trap checks, which resulted in 359 captures (251 individuals) of wood mice (*Apodemus sylvaticus*), 325 captures (149 individuals) of field voles (*Microtus agrestis*), and 257 captures (171 individuals) of bank voles (*Myodes glareolus*), totalling 941 captures (571 individuals) across the three species. Yellow-necked mice (*Apodemus flavicollis*) were excluded from all analyses as only one individual was caught. Due to the shrew escape holes, data on shrews were not recorded. In the woodland, traps that had not caught an individual previously (no previous occupant (PO) effect), caught wood mice (8.26%) more often than bank voles (3.95%) during the nocturnal period (**t**(3919) = 5.97, **p**<0.001) but, as expected from their biology, during the day bank voles (2.02%) were more likely to be caught than were wood mice (0.63%) (**t**(2261) = −3.27, **p** = 0.001). In the grassland, field voles were more likely to be caught both during the nocturnal (4.51%) (**t**(2601) = −6.53, **p**<0.001) and the diurnal (3.48%) (**t**(1467) = −6.90, **p**<0.001) periods than were wood mice (0.99%/0.07%, respectively). If the trap had a PO, there was a strong bias towards trapping same-species occupants subsequently (Fig 1). The proportion of traps capturing an animal initially (no PO) were higher during the nocturnal trapping period (woodland 12.21%/ grassland 5.50%) compared to the diurnal period (woodland 2.64% / grassland 3.55%) for both habitats (Woodland **t**(3371) = 11.68, **p**<0.001, Grassland **t**(3217) = 2.69, **p**<0.01). This held for wood mice (nocturnal 4.96% / diurnal 0.35%) (**t**(4796) = 12.76, **p**<0.001) and bank voles (nocturnal 2.30% / diurnal 1.09%) (**t**(6835) = 3.96, **p**<0.001) but not field voles (nocturnal 2.08% / diurnal 1.72%) (**t**(6445) = 1.08, **p** = 0.2824).

### Species models

**Fig 2:**
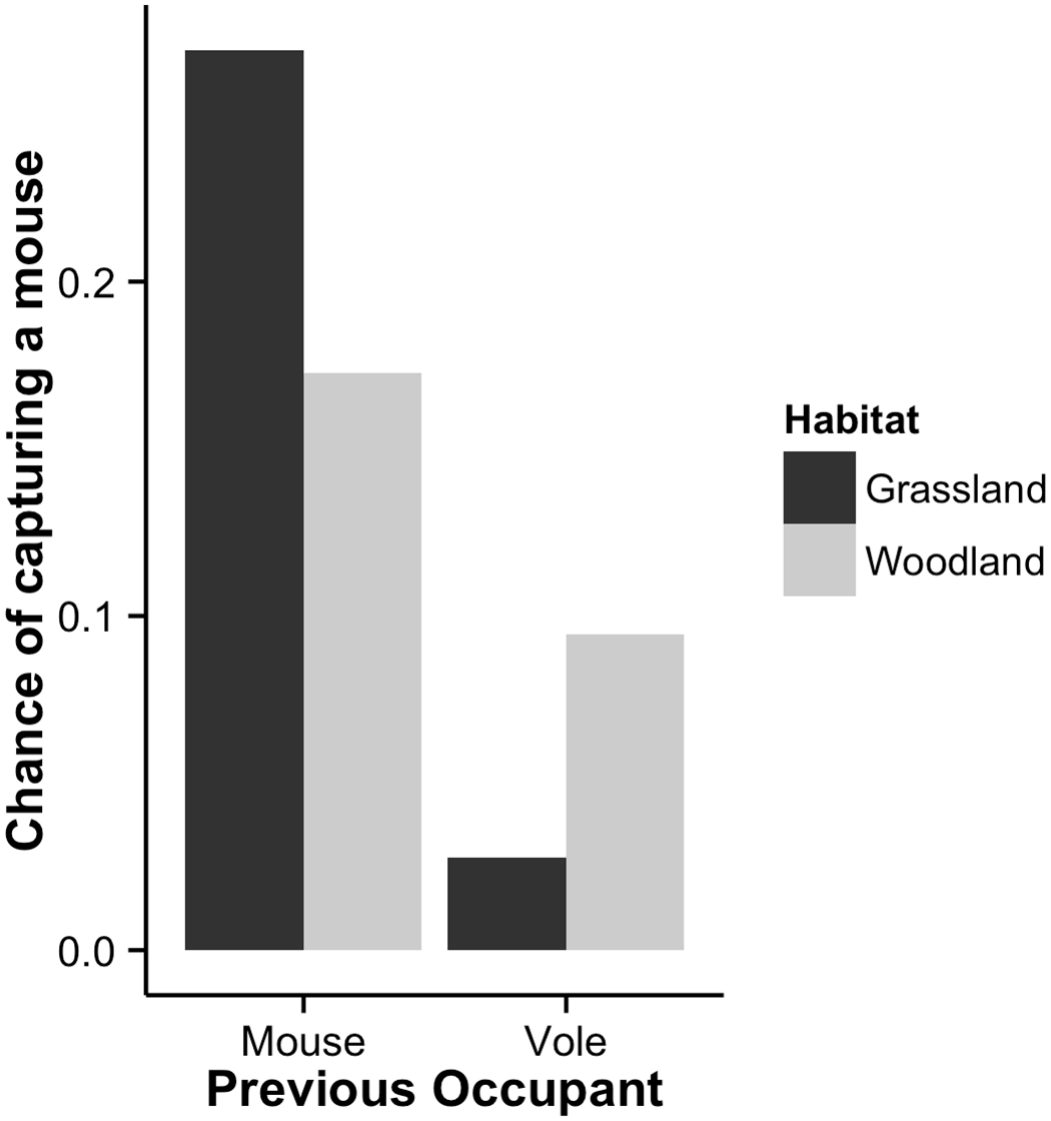
Previous Occupant, habitat interaction plot for wood mice. The interaction would suggest that the effect the PO is less pronounced in the woodland compared to the grassland Habitat. Wood mice are less likely to enter traps that previously contained field voles compared to those that contained bank voles.

A preference for conspecific POs was evident in all three models; with wood mice not being caught as frequently in vole (either bank or field vole) scented traps compared to mouse-scented traps (-3.36 ±0.61, **z** = −5.55, P<0.001) and both bank voles (1.58 ±0.24, **z** = 6.49, P<0.001) and field voles (1.36 ±0.46, **z** = 2.96, P<0.01) preferring vole-scented traps compared to mouse-scented traps. For wood mice, PO was the second most influential explanatory variable, after trapping time (diurnal or nocturnal) in explaining variance of trap success in the model; for bank voles and field voles, PO explained the most variance (Table 1). Capture rates were lower during the diurnal period compared to the nocturnal period for all three species models (wood mice: −3.43 ±0.41 **z** = −8.33, P<0.001; bank voles: −0.81 ±0.23, **z** = −3.46, P<0.001; field voles: −0.65 ±0.21, **z** = −3.10, P<0.01), as would be expected from the biology of these species. For wood mice there was an interaction between habitat and PO (Fig 2), with higher capture success when bank voles were the PO in the woodland habitat (2.41 ±0.66, **z** = 3.64, P<0.001). Estimates from the predict function inferred that same-species predominated in PO traps, with higher values during the nocturnal period compared to the diurnal period (Table 2).

**Table 1:** The explained deviance percentage for each of the covariants in the three species GLMs.

	Explained deviance %		
	Wood mice	Bank voles	Field voles
Previous Occupant	26.6	69.8	30.3
Trapping Time	54.0	15.4	9.4
Trapping Month	9.0	14.8	26.2
Habitat	5.2		
Previous Occupant / Habitat	5.2		
Trap Checks			7.6
Trap Checks since last capture			26.5

**Table 2:** Predicted Captures Probabilities from the three species GLMs.

			Capture probabilities	
Species caught	Habitat	Trapping Time	Previous mouse	Previous vole
Wood mouse	Woodland	Nocturnal	0.3229	0.1801
Bank vole	Woodland	Nocturnal	0.0750	0.2611
Wood mouse	Woodland	Diurnal	0.0226	0.0090
Bank vole	Woodland	Diurnal	0.0354	0.1445
Wood mouse	Grassland	Nocturnal	0.4882	0.0537
Field vole	Grassland	Nocturnal	0.1413	0.3166
Wood mouse	Grassland	Diurnal	0.0504	0.0020
Field vole	Grassland	Diurnal	0.0878	0.2216

### Sex models

There was a higher probability for female field voles to enter traps that contained females previously, compared to any other sex pairings (*x* ^2^(3) = 99.46, **p**<0.001). A similar trend was apparent in bank voles although the effect was slightly below significance (*x* ^2^(3) = 7.73, **p** = 0.052). Wood mice, however showed no evidence of this effect (*x* ^2^(3) = 3.62, **p** = 0.31) (S2 Table).

### Simulation

The parameters used in the simulation for the initial species capture probability were calculated from the data directly (Table 3) and from the Species models, for the PO multipliers, using the R predict function (Table 2). The values of the parameters highlighted that all traps that had a previous occupant also had a higher projected chance of capturing a SO, compared to traps that had not yet caught an animal. In the grassland there was a higher probability of projected capture for wood mice when the PO was the same species compared to no PO (49.31 times greater for diurnal period and 72 times greater for the nocturnal period (S4 Table)), if the initial capture chances for mice and voles were equal, but the sum of the species multipliers were not, then there would be a bias due to the PO effect (S1 Text).

**Table 3:** Capture probabilities.

Species caught	Habitat	Trapping Time	Capture probabilities
Wood Mouse	Woodland	Nocturnal	0.0826
Bank vole	Woodland	Nocturnal	0.0395
Wood mouse	Woodland	Diurnal	0.0063
Bank vole	Woodland	Diurnal	0.0202
Wood mouse	Grassland	Nocturnal	0.0099
Field vole	Grassland	Nocturnal	0.0451
Wood mouse	Grassland	Diurnal	0.0007
Field vole	Grassland	Diurnal	0.0348

Capture probabilities for wood mice, bank voles and field voles in the two habitats, for both time periods. The values were averaged from the raw data.

**Fig 3:**
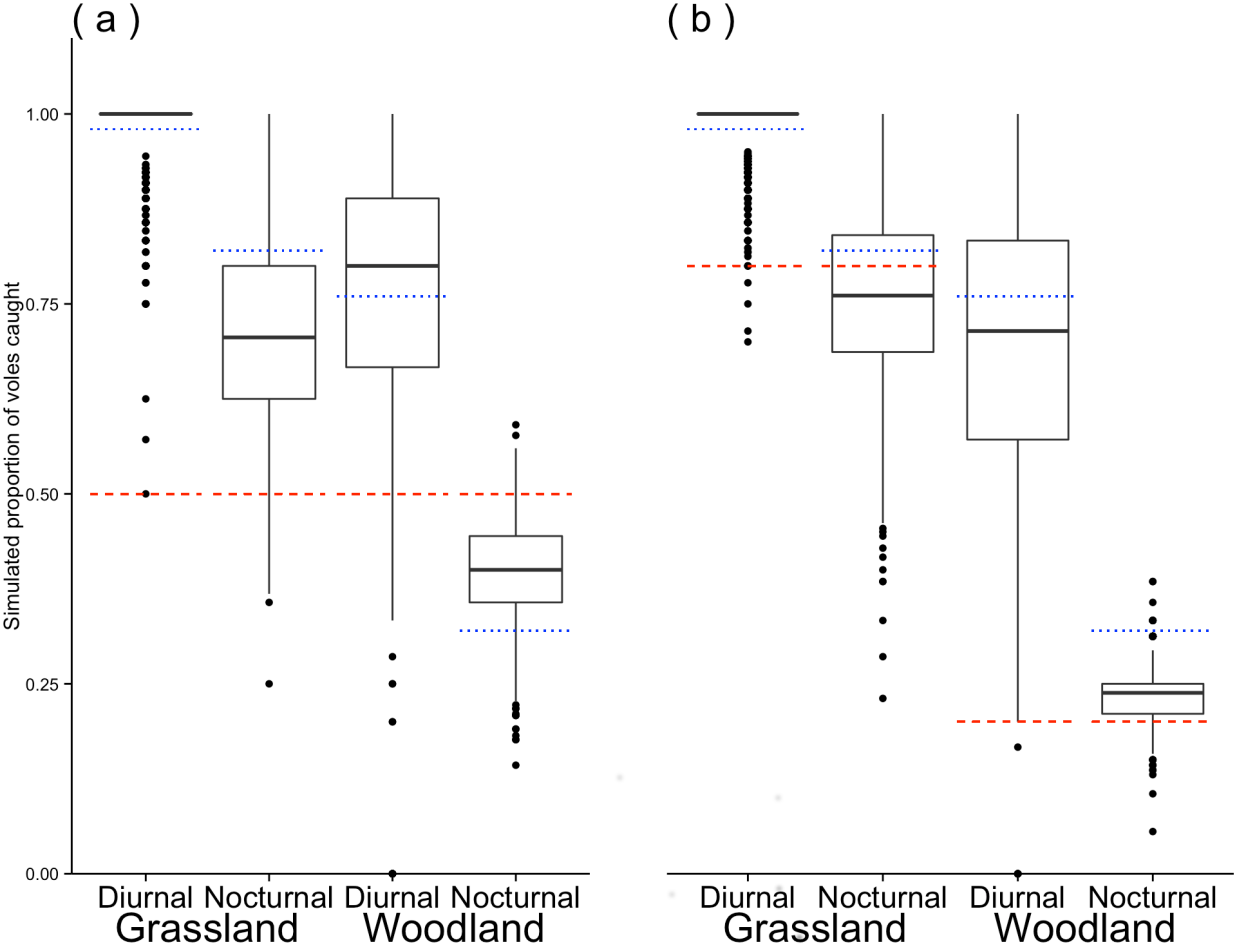
Simulated captures showing the proportion of voles caught. (a) with a 1:1 species ratio of mice to voles, (b) with a 1:4 species ratio of mice to voles in the grassland and a 4:1 ratio of mice to voles in the woodland. The red dashed line indicates the expected proportion of voles caught during the trapping simulations based just on the species ratio *E*_*v*_. The blue dotted line represents the expected proportion of voles caught during the trapping simulations based on the capture chance without any previous occupant effect *C*_*v*_. Capture proportion being defined as the number of individuals of a particular species caught divided by the total number of individuals of both species caught during the trapping session.

**Fig 4:**
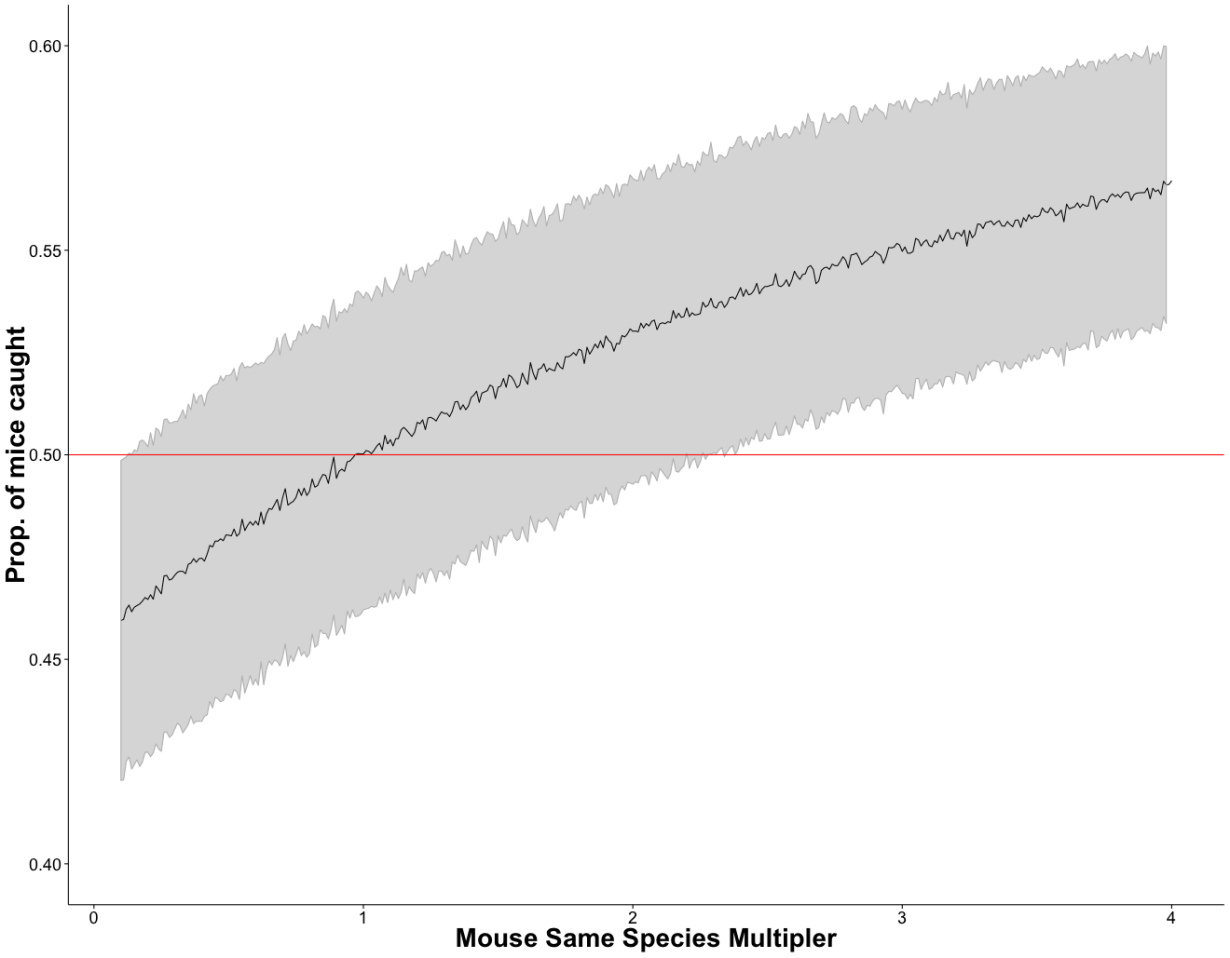
Repeat runs of simulated captures varying mouse same species multiplier. The proportion of mice caught compared to voles, for increasing values of mouse same species PO multiplier (*C*_*s*_). The initial capture chance was 20% and the other PO multipliers were set to 1.0 for both species and kept constant for all runs of the simulation.

The results from the simulation are summarised in Figs 3 and 4, S5 and S6 Tables. When simulating with equal species population densities, in the woodland nocturnal simulation fewer voles were caught than expected from the population density but more than would be expected due to capture chance alone, indicating a PO effect in favour of voles. The PO effect in favour of voles was also evident in the diurnal woodland simulation. In the grassland simulation, voles dominated both the nocturnal and diurnal periods, but field vole captures during the nocturnal period were lower than would be expected from the capture chance alone, indicating a PO effect in favour of the mice. When the simulation was set for unequal population densities (4 mice to 1 bank vole in the woodland and 1 mouse to 4 field voles in the grassland) in the woodland, during both periods there was a PO effect in favour of the mice, which was also the case during the nocturnal period in the grassland. But during the diurnal period, in the grassland, the PO effect favoured the field voles.

Repeating the simulation with an increasing mouse same species multiplier (*M*_*s*_), the number of mice caught compared to voles increased with increased values of *M*_*s*_. When all other values were equal for both species (Fig 4) it is evident that PO effect can bias population estimates. An increasing *M*_*s*_ only slightly decreased the number of voles caught but the increase in mice caught was clearly visible in the results (S1 Fig).

## Discussion

Live trapping is central to the study of small mammal population dynamics. Consequently it is important to understand any potential biases that may arise from the methods employed. The biases that result from the mechanical effectiveness of traps and their placement within the habitat have been accounted for historically by randomising the placement of the individual traps and making sure that enough traps are deployed, so that a significant proportion are left empty during the trapping [30]. Nevertheless the behavioural response to traps is rarely considered. Therefore this study focused on distinguishing the effects of species and sex on recapture rates recorded during traditional trapping studies. Previous studies have demonstrated the importance of olfactory cues in determining subsequent captures [12, 13]. The previous occupant (PO) effect, in traditional trapping data, encapsulates an individual’s prior knowledge of the trap as well as the non-random distribution of rodent species [2, 47, 48] in addition to trap-specific olfactory cues from potential other same- or different species- POs.

This study clearly shows that the PO can lead to a strong bias in subsequent captures. The significant tendency for all three species to enter traps with a same-species PO, observed in this study is in accord to what has been found previously [22]. The PO effect can be seen clearly in the simulation results, for example in the grassland habitat during the nocturnal period, vole numbers were lower than would be expected from capture chance alone. Traps with no PO had a lower capture rate in all cases. Although this may suggest strong benefits of pre-baiting to increase the capture rate, caution is required. The benefits of increased captures, at the start of trapping, have to be balanced against the potential bias that can result due to the unknown (potentially multiple) POs. Previous studies have shown a higher trappability for all three species reported in this study [49, 50], although our low capture probability may be due in part to the neophobic response to traps (in particular field voles). The high multiplier value for mouse PO effect, during the diurnal period, in the grassland also deserves consideration. Although the high value would suggest increased captures, it is still reliant on having a PO in the trap, the chance of which is low due to the low initial capture chance. The results from the simulation reflect this with nearly all captures during the diurnal period being voles. This is also correct from what we know about wood mice, which are nocturnal and rarely active during the day.

The species models and the simulation concentrate on the species level effects and did not consider additional characteristics of the individuals. Even though we had a large dataset, certain combination of characteristics were under-represented in the data and could not be analysed; we were reliant on PO providing the ‘test’ traps for subsequent captures [30]. The sex models only showed a significantly higher probability for female field voles to follow female field voles, but it is conceivable that additional factors are in effect, but could not be detected, as we could not include other characteristics. Breeding condition [33] and age [22] of both, the PO and SO have been shown to influence trap entry decisions. In addition it has been suggested that social rank [12, 16, 34] and deme membership [25] can influence the decision to enter the trap, although it is not possible to detect these influences from standard trapping data.

The trapping rates during the nocturnal and diurnal periods conformed with what is known about the activity patterns of the three species; wood mice are substantially nocturnal and voles are cathemeral, with bank voles having peaks of activity at dawn and dusk [46]. Consequently, mice were rarely caught during the day, while vole captures were almost evenly distributed between morning and evening captures. Because mice and voles both preferentially choose traps that had caught a PO of the same species, this could have a strong bearing on when researchers should put out the traps to estimate population densities; for example if traps are first deployed in the evening after sunset, population estimates will be more accurate for wood mice. As olfactory cues can remain in the traps [31] for at least as long as most trapping surveys are conducted, the effect of first trap placement time can persist throughout the whole survey.

Small mammal trapping can be employed to answer a variety of different research questions, and, the importance of the issues highlighted above will vary between them. If the study is only interested in confirming presence / absence of species in a particular area, then the PO effect may be of little importance. But if accurate population estimates are required, then it is possible that the conclusions drawn from traditional data may be inaccurate. For example it has been suggested that small mammals can be used as environmental indicators [51] of climate change or for habitat degradation and the impacts of habitat interventions, such as forestry operations. The PO effect will be particularly problematic if the aim of the study is to census multiple small mammal species in the same habitat and their interactions.

Traditional methods of estimating population densities rely on equal chances of capture. The issues highlighted above can all lead to bias in the estimates from these methods. If individuals are marked uniquely, then more advanced statistical methods such as MARK [52] or E-SURGE [53] can be used to disentangle the PO effect due to behavioural choices (for example due to the olfactory cues) from repeat captures of the same individual. Unfortunately, marking of individuals can be difficult as the standard fur clipping patterns only allow a finite number of unique marks [1] and PIT tagging individuals requires a higher level of competence. Replacing the traps that have caught an animal with a clean trap may be possible in some situations, which would remove the bias due to olfactory cues left in the traps. Nevertheless, this could potentially reduce total capture rates. Interestingly, in a previous study, no significant effects on trappability have been found due to odours left from washing the traps in a bleach solution [54]. The formalisation of the simulation suggests that, when all other factors are equal, the species with the highest PO effect will have a higher capture rate. This is in accordance with what has been suggested for the olfactory cues left in the traps [30]. The simulation also suggests that the PO effect can interact with other bias to either magnify or reduce them to a greater or lesser extent.

In summary the PO effect can bias the estimates obtained from repeat trapping and traditional population estimation methods. It is possible to detect some of the biases due to the PO effect in traditional trapping data. Using clean traps to replace traps that have caught animals can reduce the bias due to the olfactory clues, but may also reduce total capture rates. If individual marking is not practical, then designing the experiment so that enough traps are left empty during the trapping to always give individuals a choice of traps [30], may reduce (albeit not eliminate) these biases. Including additional analyses, as we have done here, may provide an idea of the strength of the PO effect, which should be taken into account in any conclusions drawn from the study. In contrast, in mark-recapture studies of uniquely marked individuals it is possible to correct for trap-happiness, trap-shyness, spatial heterogeneity in capture and survival probability, as well as covariates that can impact each of these processes. Unfortunately it is much harder to correct for these processes in systems where individuals are not identified uniquely. However, even here, these processes will still have the potential to bias results. If an in-depth knowledge of the population dynamics is required then collecting life history data for individuals at regular intervals instead of repeat trapping may be most appropriate. Collecting data in this way, allows the use of more advanced models such as Integral Projection Models (IPMs) [55]. Additionally recent advances in technology are offering promising alternative ways to estimate population densities of small mammals [56]. In the end the best methods to use will depend on the aims of the study and the resources available.

## Supporting Information

S1 Table Simulation Parameters. The values for these parameters were calculated from the raw data and from the species models using the R predict function.
(PDF)

S2 Table Test of the equality of proportions for each sex caught, depending on the previous occupant’s sex, for each of the three species. Wood mice (*Apodemus sylvaticus*), bank vole (*Myodes glareolus*) and field voles (*Microtus agrestis*). Column headers indicate the sex of the two individuals, e.g. FM = Female previous occupant, followed by a male subsequent occupant.
(PDF)

S3 Table Trap session details. Includes the habitat type the trapping was conducted in, the start date of the trapping and when the trapping concluded.
(PDF)

S4 Table Simulated scenarios. All the simulations were run 1,000 times with 50 traps over 5 trap checks. The *M*_*c*_ and *V*_*c*_ values are taken from the capture proportions and the *M*_*s*_, *M*_*d*_, *V*_*s*_ and *V*_*d*_, values are calculated from the predicted capture proportions.
(PDF)

S5 Table Expected vs simulated vole proportions. Results of the t-tests comparing the expected vole proportion *E*_*v*_ against the results from the simulations *S*_*v*_.
(PDF)

S6 Table Capture Chance vs Simulated. Results of the t-tests comparing the expected vole proportion based on the capture chance without any previous occupant effect *C*_*v*_ against the results from the simulation *S*_*v*_.
(PDF)

S1 Text Formalisation of Simulation. (PDF)

S1 Code Simulation source code. The Java source code for the simulation. Java 1.8 is required to compile and run the simulation. Please see README.TXT in root of archive for more information.
(ZIP)

S1 Fig The number of each species caught, for increasing values of mouse same species PO multiplier (*C*_*s*_). (TIFF)

S1 Data Data used in the three species GLMs. Includes SessionID, TrapID, Seq (trap check number), Month (1 to 12), Year, Habitat (Woodland or Grassland), Time (Nocturnal or Diurnal), Step (when was the last capture in this trap, 0 for no previous capture. Measured in trap checks), AsCaught (if a wood mouse was caught), MaCaught (if a field vole was caught), MgCaught (if a bank vole was caught), Prev (what was the last capture in the trap during the current trapping session. Vole, Mouse or NA).
(CSV)
