# Supplementary material for: Analysis on Population Level Reveals Trappability of Wild Rodents Is Determined by Previous Trap Occupant
Source: PLoS One. 2015 Dec 21;10(12):e0145006. doi: 10.1371/journal.pone.0145006 (PMC4687096; doi:10.1371/journal.pone.0145006)
Supplement: S1 Table — The values for these parameters were calculated from the raw data and from the species models using the R predict function. (PDF) [file pone.0145006.s001.pdf]

Table S1. Simulation Parameters

Parameter	Description
M_c	The chance of capturing a mouse in a trap with no previous occupant.
M_s	Multiplier for the mouse capture chance that determines how a previous occupant of the same species changes subsequent capture chances.
M_d	Multiplier for the mouse capture chance that determines how a previous occupant of a different species changes subsequent capture chances.
V_c	The chance of capturing a vole in a trap with no previous occupant.
V_s	Multiplier for the vole capture chance that determines how a previous occupant of the same species changes subsequent capture chances.
V_d	Multiplier for the vole capture chance that determines how a previous occupant for a different species changes subsequent capture chances.
$N(mice)$	The number of mice in the population.
$N(voles)$	The number of voles in the population.
T	The number of traps set.
T_c	The number of times that the traps were checked during the trapping.

The values for these parameters were calculated from the raw data and from the species models using the R predict function.
