# Supplementary material for: Analysis on Population Level Reveals Trappability of Wild Rodents Is Determined by Previous Trap Occupant
Source: PLoS One. 2015 Dec 21;10(12):e0145006. doi: 10.1371/journal.pone.0145006 (PMC4687096; doi:10.1371/journal.pone.0145006)
Supplement: S2 Table — Wood mice (Apodemus sylvaticus), bank vole (Myodes glareolus) and field voles (Microtus agrestis). Column headers indicate the sex of the two individuals, e.g. FM = Female previous occupant, followed by a male subsequent occupant. (PDF) [file pone.0145006.s002.pdf]

Table S2. Test of the equality of proportions for each sex caught, depending on the previous occupant's sex, for each of the three species.

Species	FF	FM	MF	MM	X-squared	df	p-value
Wood mice	0.289	0.178	0.278	0.256	3.6148	3	0.3062
Field voles	0.543	0.154	0.167	0.136	99.4568	3	< 0.001
Bank voles	0.346	0.192	0.282	0.179	7.7265	3	0.0520

Wood mice (*Apodemus sylvaticus*), bank vole (*Myodes glareolus*) and field voles (*Microtus agrestis*). Column headers indicate the sex of the two individuals, e.g. FM = Female previous occupant, followed by a male subsequent occupant.
