# Supplementary material for: Analysis on Population Level Reveals Trappability of Wild Rodents Is Determined by Previous Trap Occupant
Source: PLoS One. 2015 Dec 21;10(12):e0145006. doi: 10.1371/journal.pone.0145006 (PMC4687096; doi:10.1371/journal.pone.0145006)
Supplement: S3 Table — Includes the habitat type the trapping was conducted in, the start date of the trapping and when the trapping concluded. (PDF) [file pone.0145006.s003.pdf]

Table S3. Trap session details.

Habitat	Start Date	End Date
Woodland	2013-11-10	2013-11-12
Woodland	2013-11-13	2013-11-15
Woodland	2013-11-10	2013-11-12
Woodland	2013-11-13	2013-11-15
Woodland	2013-07-16	2013-07-18
Grassland	2002-08-04	2002-08-06
Grassland	2002-09-29	2002-10-02
Grassland	2004-07-09	2004-07-14
Grassland	2004-08-08	2004-08-11
Woodland	2003-07-14	2003-07-16
Woodland	2003-08-10	2003-08-12
Woodland	2003-07-24	2003-07-26
Woodland	2003-08-24	2003-08-27
Woodland	2003-09-28	2003-10-01
Woodland	2003-07-31	2003-08-02
Woodland	2003-09-27	2003-09-29
Woodland	2007-06-21	2007-06-23

Includes the habitat type the trapping was conducted in, the start date of the trapping and when the trapping concluded.
