# Supplementary material for: Analysis on Population Level Reveals Trappability of Wild Rodents Is Determined by Previous Trap Occupant
Source: PLoS One. 2015 Dec 21;10(12):e0145006. doi: 10.1371/journal.pone.0145006 (PMC4687096; doi:10.1371/journal.pone.0145006)
Supplement: S4 Table — All the simulations were run 1,000 times with 50 traps over 5 trap checks. The M c and V c values are taken from the capture proportions and the M s, M d, V s and V d, values are calculated from the predicted capture proportions. (PDF) [file pone.0145006.s004.pdf]

Table S4. Simulated scenarios.

	Parameters							
Scenario	M_c	M_s	M_d	V_c	V_s	V_d	M	V
Woodland Nocturnal, wood mice 1:1 bank voles	0.0826	3.91	2.18	0.0395	6.61	1.90	20	20
Woodland Diurnal, wood mice 1:1 bank voles	0.0063	3.59	1.43	0.0202	7.15	1.75	20	20
Grassland Nocturnal, wood mice 1:1 field voles	0.0099	49.31	5.42	0.0451	7.02	3.13	20	20
Grassland Diurnal, wood mice 1:1 field voles	0.0007	72.00	2.86	0.0348	6.37	2.52	20	20
Woodland Nocturnal, wood mice 4:1 bank voles	0.0826	3.91	2.18	0.0395	6.61	1.90	20	5
Woodland Diurnal, wood mice 4:1 bank voles	0.0063	3.59	1.43	0.0202	7.15	1.75	20	5
Grassland Nocturnal, wood mice 1:4 field voles	0.0099	49.31	5.42	0.0451	7.02	3.13	20	80
Grassland Diurnal, wood mice 1:4 field voles	0.0007	72.00	2.86	0.0348	6.37	2.52	20	80

All the simulations were run 1,000 times with 50 traps over 5 trap checks. The M_c and V_c values are taken from the capture proportions and the M_s, M_d, V_s and V_d, values are calculated from the predicted capture proportions.
