# Supplementary material for: Analysis on Population Level Reveals Trappability of Wild Rodents Is Determined by Previous Trap Occupant
Source: PLoS One. 2015 Dec 21;10(12):e0145006. doi: 10.1371/journal.pone.0145006 (PMC4687096; doi:10.1371/journal.pone.0145006)
Supplement: S5 Table — Results of the t-tests comparing the expected vole proportion E v against the results from the simulations S v. (PDF) [file pone.0145006.s005.pdf]

Table S5. Expected vs simulated vole proportions.

Scenario	E_v	S_v	S_v SE	t-test results			
Woodland Nocturnal, wood mice 1:1 bank voles	0.5	0.40	0.002	t(999) =	−48.17	**p** =	< 0.001
Woodland Diurnal, wood mice 1:1 bank voles	0.5	0.78	0.006	t(999) =	50.47	**p** =	< 0.001
Grassland Nocturnal, wood mice 1:1 field voles	0.5	0.71	0.004	t(999) =	54.77	**p** =	< 0.001
Grassland Diurnal, wood mice 1:1 field voles	0.5	0.98	0.002	t(999) =	271.20	**p** =	< 0.001
Woodland Nocturnal, wood mice 4:1 bank voles	0.2	0.23	0.001	t(999) =	30.39	**p** =	< 0.001
Woodland Diurnal, wood mice 4:1 bank voles	0.2	0.72	0.006	t(999) =	83.55	**p** =	< 0.001
Grassland Nocturnal, wood mice 1:4 field voles	0.8	0.76	0.004	t(999) =	−10.55	**p** =	< 0.001
Grassland Diurnal, wood mice 1:4 field voles	0.8	0.98	0.001	t(999) =	129.68	**p** =	< 0.001

Results of the t-tests comparing the expected vole proportion E_v against the results from the simulations S_v. Here vole proportion is defined as the number of unique voles caught divided by the number of individuals of both species caught during the trapping session.
