# Supplementary material for: Analysis on Population Level Reveals Trappability of Wild Rodents Is Determined by Previous Trap Occupant
Source: PLoS One. 2015 Dec 21;10(12):e0145006. doi: 10.1371/journal.pone.0145006 (PMC4687096; doi:10.1371/journal.pone.0145006)
Supplement: S6 Table — Results of the t-tests comparing the expected vole proportion based on the capture chance without any previous occupant effect C v against the results from the simulation S v. (PDF) [file pone.0145006.s006.pdf]

Table S6. Results of the t-tests comparing the expected vole proportion based on the capture chance without any previous occupant effect C_v against the results from the simulation S_v.

Scenario	C_v	$\overline{S_V}$	$\overline{S_v}$ SE	t-test results			
Woodland Nocturnal, wood mice 1:1 bank voles	0.32	0.40	0.002	t(999) =	35.91	**p** =	< 0.001
Woodland Diurnal, wood mice 1:1 bank voles	0.76	0.78	0.006	t(999) =	3.82	**p** =	0.001
Grassland Nocturnal, wood mice 1:1 field voles	0.82	0.71	0.004	t(999) =	−27.33	**p** =	< 0.001
Grassland Diurnal, wood mice 1:1 field voles	0.98	0.98	0.002	t(999) =	−1.85	**p** =	0.06
Woodland Nocturnal, wood mice 4:1 bank voles	0.32	0.23	0.001	t(999) =	−80.36	**p** =	< 0.001
Woodland Diurnal, wood mice 4:1 bank voles	0.76	0.72	0.006	t(999) =	−7.04	**p** =	3.53
Grassland Nocturnal, wood mice 1:4 field voles	0.82	0.76	0.004	t(999) =	−15.99	**p** =	< 0.001
Grassland Diurnal, wood mice 1:4 field voles	0.98	0.98	0.001	t(999) =	0.79	**p** =	0.43
