# Supplementary material for: Analysis on Population Level Reveals Trappability of Wild Rodents Is Determined by Previous Trap Occupant
Source: PLoS One. 2015 Dec 21;10(12):e0145006. doi: 10.1371/journal.pone.0145006 (PMC4687096; doi:10.1371/journal.pone.0145006)
Supplement: S1 Text — (PDF) [file pone.0145006.s007.pdf]

The simulation can formalised as follows. To avoid invalid capture chances the following restrictions were placed on the parameters:

$M_c + V_c$ must be between 0 and 1.

$(M_c \cdot M_s) + (V_c \cdot V_d)$ must be between 0 and 1.

$(M_c \cdot M_d) + (V_c \cdot V_s)$ must be between 0 and 1.

The following variables are used within the simulation.

Parameter	Description
$n(mice)$	The number of available mice, not currently caught in a trap.
$n(voles)$	The number of available voles, not currently caught in a trap.
$n(micecaught)$	The number of individual mice caught during the trapping.
$n(volescaught)$	The number of individual voles caught during the trapping.

We introduce random variables

$$c = \begin{cases} m & \text{mice caught} \\ v & \text{vole caught} \\ 0 & \text{nothing caught} \end{cases}$$

and

$$p = \begin{cases} m & \text{mice caught previously} \\ v & \text{vole caught previously} \\ 0 & \text{nothing caught previously} \end{cases}$$

We calculate that

$$P(c = m|p = 0) = \frac{M_c \cdot n(mice)}{N(mice)}$$

$$P(c = v|p = 0) = \frac{V_c \cdot n(voles)}{N(voles)}$$

$$P(c = 0|p = 0) = 1 - \left(\frac{M_c \cdot n(mice)}{N(mice)} + \frac{V_c \cdot n(voles)}{N(voles)}\right)$$

$$P(c = m|p = m) = \frac{M_c \cdot M_s \cdot n(mice)}{N(mice)}$$

$$P(c = v|p = m) = \frac{V_c \cdot V_d \cdot n(voles)}{N(voles)}$$

$$P(c = 0|p = m) = 1 - \left(\frac{M_c \cdot M_s \cdot n(mice)}{N(mice)} + \frac{V_c \cdot V_d \cdot n(voles)}{N(voles)}\right)$$

$$P(c = m|p = v) = \frac{M_c \cdot M_d \cdot n(mice)}{N(mice)}$$

$$P(c = v|p = v) = \frac{V_c \cdot V_s \cdot n(voles)}{N(voles)}$$

$$P(c = 0|p = v) = 1 - \left(\frac{M_c \cdot M_d \cdot n(mice)}{N(mice)} + \frac{V_d \cdot V_s \cdot n(voles)}{N(voles)}\right)$$

We can concluded that, if there is no difference between the capture chance for mice M_c and voles V_c, then if

$$(M_d + M_s) \neq (V_d + V_s)$$

there will be a bias in the capture rates due to the previous occupant effect.
