# Supplementary figures and images for: Analysis on Population Level Reveals Trappability of Wild Rodents Is Determined by Previous Trap Occupant
Source: PLoS One. 2015 Dec 21;10(12):e0145006. doi: 10.1371/journal.pone.0145006 (PMC4687096; doi:10.1371/journal.pone.0145006)

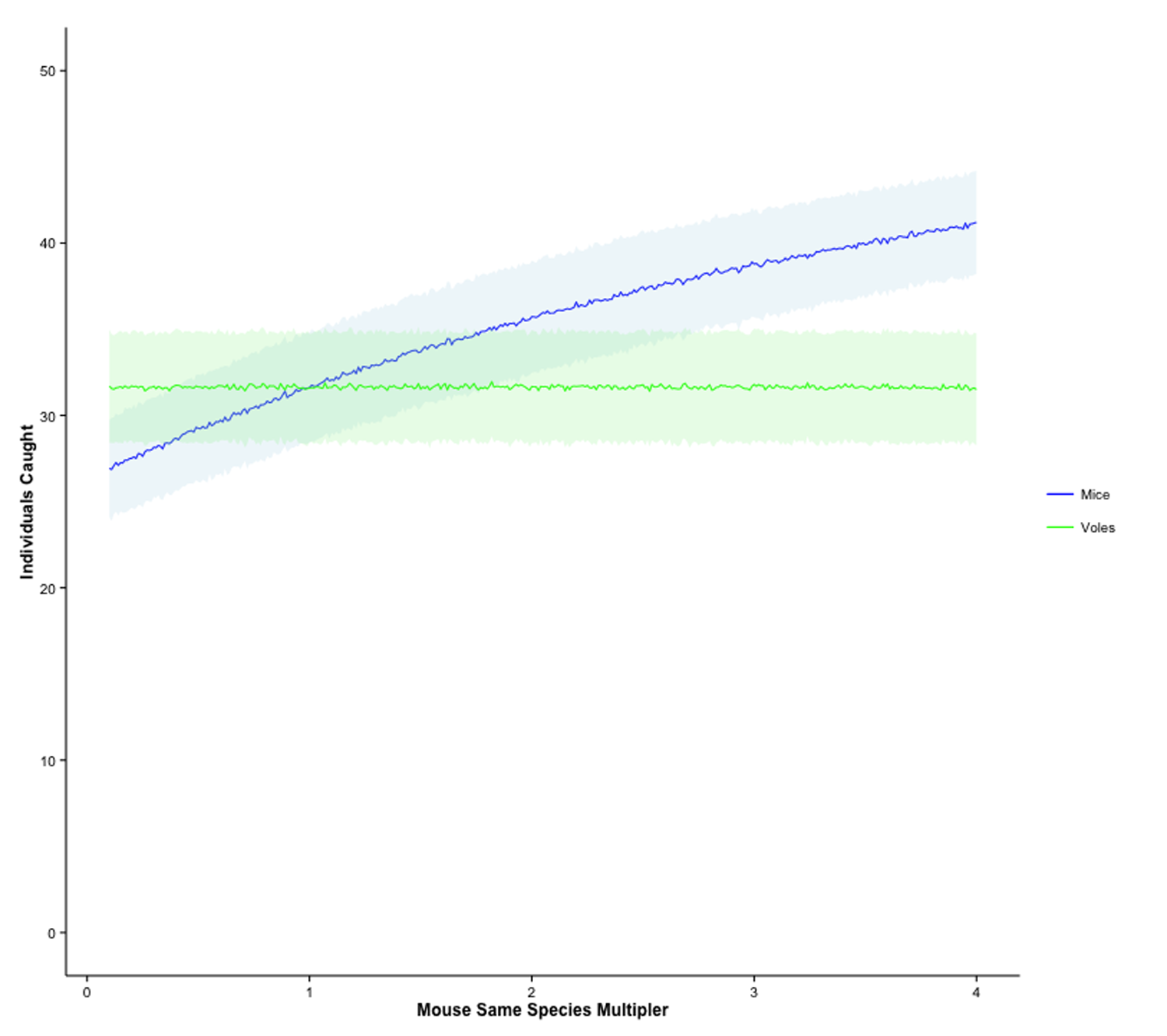

Supplement: S1 Fig — (TIFF) [file pone.0145006.s009.tiff]
